# Therapeutic Use of Exercising in Hypoxia: Promises and Limitations

**DOI:** 10.3389/fphys.2016.00224

**Published:** 2016-06-10

**Authors:** Gregoire P. Millet, Tadej Debevec, Franck Brocherie, Davide Malatesta, Olivier Girard

**Affiliations:** ^1^Institute of Sport Sciences of the University of Lausanne (ISSUL)Lausanne, Switzerland; ^2^Department of Automation, Biocybernetics and Robotics, Jožef Stefan InstituteLjubljana, Slovenia

**Keywords:** hypoxia-inducible factor 1, alpha subunit, hypoxia, brain, obesity, hypertension, elderly

It is well-established that different altitude training modalities can improve convective oxygen (O_2_) transport capacity and physical fitness of athletes (Millet et al., 2010). Exercising in hypoxia also induces specific muscular adaptations including increased oxidative enzymes (e.g., citrate synthase) activity, mitochondrial density, capillary-to-fiber ratio, and fiber cross-sectional area (Hoppeler et al., 2008). These changes with hypoxic training are mostly modulated via hypoxia-inducible factor 1α (HIF-1α) signaling cascade, which is not activated to the same extent when training is performed in normoxia or by passive hypoxic exposure. Indeed, large body of literature shows that, compared to hypoxic exercise, passive exposure to hypoxia does not provoke similar acute responses. In healthy individuals, both systemic (e.g., performance enhancement), cardiovascular (e.g., maximal O_2_ uptake, VO_2max_) or transcriptional muscular responses are minimal with intermittent passive exposures at moderate altitude. On the other hand, there are clear evidences that when hypoxia is combined with exercise, it triggers specific responses, not observed following similar exercise in normoxia (Bartsch et al., 2008; Lundby et al., 2009). In addition, greater specific adaptations have been reported in high-intensity vs. moderate-intensity hypoxic intervention (Faiss et al., 2013) (e.g., improvements in muscle O_2_ homeostasis and tissue perfusion induced by enhanced mitochondrial efficiency, control of mitochondrial respiration, angiogenesis, and muscle buffering capacity). It seems that the main underlying mechanism is the larger hypoxemia resulting from the combination of muscle deoxygenation (high-intensity exercise) and systemic desaturation (moderate hypoxia).

In patients or elderly individuals, altitude is generally associated with increased health risks through enhanced sympathetic vasoconstrictor activation (Blitzer et al., 1996), obstructive sleep apneas (Nespoulet et al., 2012), hypoxemia (Levine et al., 1997), pulmonary hypertension (Valencia-Flores et al., 2004), arrhythmias (Kujanik et al., 2000), and alterations of postural control (Degache et al., 2012). However, several studies have investigated the therapeutic benefits of exercising in mild hypoxia on the blood pressure regulation and the influence of different hypoxic modalities in healthy individuals (Bailey et al., 2001; Wang et al., 2007; Haufe et al., 2008; Nishiwaki et al., 2011; Morishima et al., 2014; Shi et al., 2014) or in patients with different cardiovascular and respiratory risk factors such as chronic obstructive pulmonary disease (COPD) (Haider et al., 2009), obesity (Wiesner et al., 2010), coronary artery disease (Burtscher et al., 2004). Recent studies (Haufe et al., 2008; Wiesner et al., 2010) have also reported that sustained hypoxia may be of benefit to weight management programs of obese patients (Urdampilleta et al., 2012; Kayser and Verges, 2013). Both exercise (Williams et al., 2002) and/or intermittent hypoxia (Burtscher et al., 2004; Shatilo et al., 2008) have been suggested to positively influence age-related alterations in elderly individuals. Finally, living at altitude seems to have contradictory effects on different mortality risk factors.

Therefore, this essay summarizes recent evidences suggesting that exercising in hypoxia might be a valuable and viable “therapeutic strategy.” We discuss the benefits and risks/limitations in (i) hypertensive (ii) obese, (iii) elderly individuals. Since the benefits of being active have been extensively investigated in these three groups of individuals (see respective reviews on the effects of physical activity in Cherubini et al., 1998; Baillot et al., 2014; Borjesson et al., 2016), the present article focus on the potential additional health benefits provided by hypoxic exercise, when compared to normoxic exercise. For safety and practical reasons, patients cannot access high altitude (even by using hypoxic devices) and preferably stay at moderate altitude (1800–3000 m). In this setting, exercise is used to increase the overall hypoxia-induced metabolic stress and thereby provide benefits beyond those achievable by normoxic therapeutic training modalities.

## Hypertension

Systemic hypoxia (i.e., reduction in the O_2_ arterial content) at rest elicits acute vasodilation in conduit arteries [reduction in arterial stiffness (Vedam et al., 2009)] and augments blood flow within the skeletal muscle vascular beds, which occurs despite an enhanced sympathetic vasoconstrictor activity. There are direct evidences that during hypoxic exposure endothelium-derived nitric oxide (NO)-mediated mechanisms are largely involved in the vasodilatation of muscular arteries (but not the aorta; Vedam et al., 2009). Leuenberger et al. (2008) further showed that hypobaric hypoxia is associated with increased NO in venous effluent from skeletal muscle but not in the skeletal muscle interstitium.

Systemic hypoxia *per se* leads to peripheral vasodilation that aims to counteract the decrease in O_2_ content and subsequent peripheral O_2_ delivery. In former Soviet Union countries, intermittent hypoxic exposure at rest (Bernardi et al., 2001) was applied therapeutically to lower blood pressure in hypertensive patients with numerous positive reports (Serebrovskaya et al., 2008). Skeletal muscle vasodilation associated with hypoxia is due to release of vasodilator substances of which the NO/NO synthase pathway seems to play a central role. However, there is a synergistic effect of hypoxic and exercise stressors on the magnitude of this response. When physical exercise is added to the hypoxic exposure, blood flow increases to contracting muscles that compensate for the reduced arterial O_2_ content and keeps O_2_ delivery to the active muscle relatively constant; a phenomenon called “compensatory vasodilatation” (Casey and Joyner, 2011). Although, a number of other vasoactive substances are also produced by the endothelium in an O_2_-sensitive manner [i.e., adenosine (Leuenberger et al., 1999); prostaglandins (Messina et al., 1992)], NO appears to be the major contributor to the compensatory vasodilator responses. Hypoxia and physical exercise are independent and highly potent metabolic stressors (Bailey et al., 2001). Acute hypoxic exposure reduces arterial O_2_ saturation level, whereas physical exercise increases VO_2max_ by working muscles (Wang et al., 2010). Hence, hypoxic exercise considerably reduces the O_2_ partial pressure within the mitochondria of the working organs by simultaneously decreasing O_2_ supply and increasing O_2_ demand (Wang et al., 2010). Owing to hypoxia-induced augmentation of exercise hypoxemia, the reduction of blood pressure by “compensatory vasodilatation” mechanisms may be larger than that by exercise alone. In particular, compared to similar training at sea level, moderate-intensity hypoxic training was shown to be superior for improving aerobic capacity and increasing the production of various circulating progenitor cells subsets and angiogenic factors, associated with enhanced hemodynamic responses to exercise in sedentary individuals (i.e., vasodilation in coronary and resistance vessels by increased NO production, vascular endothelial growth factor (VEGF) production; Wang et al., 2007). Similarly, exercise training under mild intermittent hypoxic conditions (2000 m simulated altitude) seems to be more efficient in reducing arterial stiffness and inducing vascular functional adaptation in the form of an increased flow mediated dilation (FMD) response amplitude compared to similar normoxic training (Nishiwaki et al., 2011). Taken together the above evidences suggest that adding hypoxic stimuli to exercise induces greater reductions in blood pressure, compared to normoxic training and enhances various aspects of the vascular function, which are pertinent for the reduction of cardiovascular risks.

## Obesity

Obesity is characterized by an increased mass of adipose tissue (excessive fat accumulation) resulting from a systemic imbalance between food intake and energy expenditure (i.e., positive energy balance). Beside obesity-related disorders, the visceral adiposity impairs endothelial function and increases the vascular media thickness and arterial stiffness (Weil et al., 2011). Obese individuals often present chronic inflammation of the adipose tissue, which is considered to play an important role in the initiation and development of obesity-related comorbidities, and increases of the oxidative stress within the fat tissue. Hypoxia seems to be involved in the inflammation-related within the poorly oxygenated adipose tissue (e.g., secretion of several adipokines affecting endothelial function and promoting a systemic inflammatory state). Thus, systemic and local inflammation associated with oxidative stress, adipokine dysregulation and increased sympathetic nervous actions is implicated in endothelial dysfunction in obesity.

Pioneering studies (Haufe et al., 2008; Wiesner et al., 2010) have reported that, despite lower training workload and therefore lower mechanical strain in hypoxia, this environmental condition may lead to significant weight loss and improve metabolic and cardiorespiratory health, leading to suggestions that sustained hypoxia may be of benefit to weight management programs of obese patients (Urdampilleta et al., 2012; Kayser and Verges, 2013). This phenomenon is also known as “altitude anorexia” and is underlined by decreased food intake and hypoxia-induced appetite reduction, as a consequence of increases in the appetite reducing and satiety signaling peptides leptin or cholecystokinin, concomitant with a reduction in the hunger-stimulating hormone ghrelin (Yingzhong et al., 2006). In addition, hypoxia exposure also seems to result in increased energy expenditure (Kayser and Verges, 2013). In line with the above, higher altitude of residence was recently shown to be associated to lower obesity rates (Voss et al., 2014).

## Aging

Aging is associated with a progressive increase in systolic blood pressure and development of arterial hypertension (via atherosclerotic changes, stiffening of arteries, altered renal function, and arterial baroreflex impairment), leading to increased risk for cardiovascular or coronary heart diseases (Levy et al., 1996). Moreover, sarcopenia (muscle loss) is accompanying aging (Janssen et al., 2002), mainly due to a decrease in fast-twitch fiber cross-sectional area (Verdijk et al., 2007). This phenomenon may be masked by fat mass accumulation (Gallagher et al., 2000), which also reduces vascularization and angiogenic capacity and increases the risk of cardio-metabolic disorders. Overall, these degenerative-dystrophic alterations predispose tissue to local hypoxia (Lenaz et al., 2002; Sharma and Goodwin, 2006). However, altitude residence' effect appears controversial. For instance, it may detrimentally affect certain lung conditions such as chronic COPD (Cote et al., 1993) or pneumonia (Perez-Padilla and Franco-Marina, 2004). On the other hand, certain cardiovascular risks seem to be reduced with living in altitude. In particular lower ischemic heart disease risk (Faeh et al., 2009, 2016) and reduction in mortality from coronary heart disease (Mortimer et al., 1977) or dialysis (Winkelmayer et al., 2009; Shapiro et al., 2014) have been reported in high altitude patients. Improved myocardial angiogenesis or ventricular remodeling have been proposed as the main underlying mechanisms (Sasaki et al., 2002).

While caution may be requested regarding the utilization of hypoxic training with elderly individuals, passive intermittent hypoxic exposure was shown to increase exercise tolerance and VO_2max_ (Burtscher et al., 2004). Furthermore, healthy elderly individuals well-tolerated intermittent hypoxic training, with greater effect on haemodynamic, microvascular endothelial function, and work capacity in untrained participants (Shatilo et al., 2008). Recently, resistance training under systemic hypoxia was shown to result in greater muscle size and strength and endurance increases as well as angiogenesis in the skeletal muscles (Kon et al., 2015). Taken together, these findings suggest that combining exercise, be it low- to-high-intensity aerobic or resistance, with hypoxic stressor would play a role in slowing sarcopenia development as well as improving physical capacity (via hypotensive and antioxidant actions) and well-being of elderly individuals.

Logically, the next question is: what is the optimal combination of exercise and hypoxia?

Hypoxia and exercise may have synergistic (positive) effects in hypertensive, obese or elderly subjects. However, little is known regarding the optimal combination between the physical activity (e.g., exercise intensity, type of activity) and hypoxic (e.g., altitude level, optimal hypoxic dose, normobaric vs. hypobaric hypoxia) components. Different combinations of these two factors have to be further investigated to identify optimal and individually tailored hypoxic exercise regimens. Based on the provided evidences such protocols could result in (i) reduction of metabolic and cardiovascular risk factors primarily related to improved vascular function (NO bioavailability) in addition to positive muscular (up-regulation of muscle oxidative enzymes, ion transport proteins and muscle activation/perfusion) and (ii) neuro-vegetative adaptations.

## Author contributions

All authors listed, have made substantial, direct and intellectual contribution to the work, and approved it for publication.

### Conflict of interest statement

The authors declare that the research was conducted in the absence of any commercial or financial relationships that could be construed as a potential conflict of interest.
